# Accuracy of Rapid Tests for Malaria and Treatment Outcomes for Malaria and Non-Malaria Cases among Under-Five Children in Rural Ghana

**DOI:** 10.1371/journal.pone.0034073

**Published:** 2012-04-13

**Authors:** Frank Baiden, Jayne Webster, Mathilda Tivura, Rupert Delimini, Yvonne Berko, Seeba Amenga-Etego, Akua Agyeman-Budu, Akosua B. Karikari, Jane Bruce, Seth Owusu-Agyei, Daniel Chandramohan

**Affiliations:** 1 Malaria Group, Kintampo Health Research Centre, Kintampo, Ghana; 2 Department of Disease Control, London School of Hygiene and Tropical Medicine, London, United Kingdom; 3 Computer Centre, Kintampo Health Research Centre, Kintampo, Ghana; 4 Clinical Laboratory, Kintampo Health Research Centre, Kintampo, Ghana; 5 Microbiology Department, University of Development Studies, Tamale; 6 Directorate, Kintampo Health Research Centre, Kintampo, Ghana; Menzies School of Health Research, Australia

## Abstract

**Background:**

WHO now recommends test-based management of malaria across all transmission settings. The accuracy of rapid diagnostic test (RDT) and the outcome of treatment based on the result of tests will influence acceptability of and adherence to the new guidelines.

**Method:**

We conducted a study at the Kintampo hospital in rural Ghana to evaluate the performance of CareStart, a HRP-2 based RDT, using microscopy as reference. We applied IMCI treatment guidelines, restricted ACT to RDT-positive children and followed-up both RDT-positive (malaria) and RDT-negative (non-malaria) cases over 28 days.

**Results:**

436 children were enrolled in the RDT evaluation and 391 (children with haemoglobin >8.0 gm/dl) were followed-up to assess treatment outcomes. Mean age was 25.4 months (s.d. 14.6). Sensitivity and specificity of the RDT were 100.0% and 73.0% respectively. Over the follow-up period, 32 (18.5%) RDT-negative children converted to positive, with 7 (4.0%) of them presenting with fever. More children in the non-malaria group made unscheduled visits than children in the malaria group (13.3% versus 7.7%) On all scheduled follow-up visits, proportion of children having a temperature higher than that recorded on day 0 was higher in the non-malaria group compared to the malaria group. Reports of unfavourable treatment outcomes by caregivers were higher among the non-malaria group than the malaria group.

**Conclusions:**

The RDT had good sensitivity and specificity. However a minority of children who will not receive ACT based on RDT results may develop clinical malaria within a short period in high transmission settings. This could undermine caregivers' and health workers' confidence in the new guidelines. Improving the quality of management of non-malarial febrile illnesses should be a priority in the era of test-based management of malaria.

**Trial Registration:**

ClinicalTrials.gov NCT00832754

## Introduction

IMCI guidelines promoted the presumptive treatment of malaria for all febrile illnesses in children in high transmission settings. [1], [2] This approach contributed to over-diagnosis and over-emphasis on malaria to the neglect of other causes of childhood febrile illnesses [3], [4], [5], [6], [7]. Chloroquine and sulphadoxine-pyrimethamine, the cheap and safe antimalarial drugs that justified the presumptive approach are no longer effective. Since they are now replaced with the more expensive artemisinin-based combination therapy (ACT), there has been a move towards the targeted use of the ACTs [8], [9], [10], [11]. The World Health Organisation (WHO) has revised malaria treatment guidelines, restricting the use of ACT to only parasitologically-confirmed cases [12]. As availability of microscopy is very limited in most places in sub-Saharan Africa, rapid diagnostic tests (RDTs) are the means through which the confirmation of malaria diagnosis will be achieved. The accuracy, reliability and outcome of treatment based on results of RDT will be a major determinant of the acceptability and adherence to the new guidelines. [13], [14].

The use of RDT would more clearly delineate non-malaria febrile illnesses and it is argued that this will lead to improved management of such cases [15], [16], [17], [18], [19], [20]. Conversely, RDTs are not perfect tools and false results could undermine the confidence of clinicians and lead to a lack of adherence to the new guidelines.[21], [22] False-negative results present the greater challenge as they may lead to delay in the initiation of treatment and possible deterioration in the condition of the child.[7].

As the incidence of malaria declines, increasingly more children will present to health facilities with low levels of parasitaemia. At low levels of parasitaemia, the probability of a false-negative result is higher and this could cause delay in the initiation of treatment. On the other hand, in high transmission settings a positive RDT result does not necessarily rule out bacterial or viral co-infections, as symptomatic malaria parasitaemia is not uncommon. [23], [24]

In the era of presumptive treatment, nearly all children who presented with febrile illness received an antimalarial. In addition to clearing parasites in children with malaria, this “opportunistic presumptive treatment” probably prevented new malaria infections through post treatment prophylaxis in all children. It is hypothesised that restricting ACTs to RDT-positive cases would lead to increased frequency of malaria in children since a large number of children would be denied this inadvertent prophylactic benefit and make them prone to malaria sooner and more frequently than children who receive antimalarials. [25]

The over-emphasis on malaria over the years has been at the expense of attention to non-malarial febrile illnesses. Very little is known about the aetiology and epidemiology of non-malaria fevers in sub-Saharan Africa. [26] With the use of RDTs, a substantial proportion of febrile illness in children will likely be diagnosed as non-malaria fevers. The treatment outcome in this group of children, who will be denied ACT, is likely to influence the acceptability of the new policy by health workers and caregivers who are used to receiving antimalarial prescriptions for all febrile illness in children.

In Ghana, the management of fever in under-five children has for many years been based on guidelines in the Integrated Management of Childhood Illnesses (IMCI). [1] The Ghana National Malaria Control Programme (NMCP) is in the process of revising the national guidelines for the treatment of malaria based on the revised WHO recommendations. The confirmation of malaria in Ghana will be done by RDTs as microscopy is still not widely available. Several HRP-2 based RDTs including CareStart *p.f* are being considered for roll out nationally. Studies in Africa show considerable variability in sensitivity and specificity of HRP 2 antigen detection tests. [20], [26], [27] There is also very limited evidence on the clinical outcomes of febrile children managed on the basis of RDT results. We therefore conducted a prospective cohort study to evaluate the accuracy and reliability of CareStart *p.f.* for the diagnosis of malaria in Ghana, and the treatment outcome in both RDT positive (malaria) and RDT negative (non-malaria) cases. We evaluated CareStart p.f. because it was the RDT that the NMCP was considering deploying in the country at the time of initiating this study.

## Methods

The protocol for this study is available as supporting information; see Protocol S1.

### Study site

The study was conducted in Kintampo North municipality of Ghana which lies within the forest-savannah transition belt and has malaria transmission all year round. The entomological inoculation rate was estimated at 269 infective bites per person per year. Peak incidence of malaria (8.6 per child per year) occurs in children aged 12–35 months. [28], [29] The study was undertaken in the out-patients department (OPD) of the Kintampo Municipal Hospital from February 2009 to February 2010.

### Study procedures

Caregivers residing within 15 km of the hospital who presented with children aged 3–60 months, with complaints that included fever, either on presentation (temperature>37.5°C) or within the previous 48 hours, were requested to take part in the study. After individual informed consent had been obtained, 50 µg of capillary blood was taken from all children to estimate haemoglobin using Hemocue^TM^, perform rapid test for malaria using CareStart^TM^, and prepare thin and thick blood smears for microscopy. Filter paper samples were taken for PCR analysis. Children were excluded if they had any of the IMCI danger signs or other signs of severe disease. Children with Hb<8 gm/dl were also excluded as a precautionary measure given uncertainty about the accuracy of the RDT and the practice of restricting ACT to RDT-positive cases at the time of initiating the study. Children were also ineligible if they were enrolled in other clinical studies. Children were enrolled as and when they presented at the clinic and were found to be eligible. This occurred within normal OPD working hours (0800–1400 hrs), Mondays to Fridays.

All the children were assessed by a study physician using a standard morbidity assessment questionnaire that ensured comprehensive IMCI-based case management. After clinical assessment, 5 mls of venous blood was taken under aseptic conditions for blood culture. Malaria was diagnosed and treated with an ACT only when RDT was positive. Other medications were prescribed according to identified co-morbidities. RDT-negative cases were managed on the basis of the conditions that were diagnosed using IMCI case management guidelines.

The first doses of all prescribed medications were administered under direct observation by a study nurse. Caregivers were educated on the correct procedure for administering the remaining medications at home. The follow-up schedule and the procedures were explained to caregivers prior to leaving the facility. All children (irrespective of RDT results) were followed-up in the clinic on days 1, 2, 3, 7, 14 and 28 post-treatment. Caregivers who failed to show up by mid-day of a scheduled appointment were followed-up to their places of residence. The reason for non-attendance was ascertained and assistance was offered to enable attendance at the clinic within 24 hours. Caregivers were reimbursed for their transportation costs. On each follow-up visit, a standard morbidity questionnaire was administered by a trained research assistant. Caregivers were asked to indicate whether they considered the condition of the child to be “improving”, “unchanged”, “worsening” or with “new illness”. On days 1, 7, 14, 28 post-treatment and on all unscheduled visits, blood sample was taken for RDT and blood smear microscopy. Whenever a caregiver reported “unchanged”, “worsening” or “new illness”, the child was further assessed by the study clinician.

Notwithstanding the scheduled appointments, caregivers were advised to bring their children to the hospital at any time that the condition of the child did not improve or when new symptoms developed (unscheduled visits).

### Sample size

The primary end point for estimating the sample size was the sensitivity of RDTs to diagnose malaria. We assumed that the RDTs should have at least 95% sensitivity to be useful. To estimate a sensitivity of 95% with a 95% confidence limit of +/− 3%, 203 true positive cases of malaria were required. Our second assumption was that the proportion of participants clearing fever by day 3 post-treatment would be 95% in the RDT positive cohort and 85% in the RDT negative cohort. The loss to follow up by day 3 would be <5%. To have 80% power to detect this difference between the two cohorts with 95% significance, 170 (159 plus allowance for loss to follow-up) participants were needed in each cohort.

### Laboratory analysis

All blood smear microscopy was performed at the clinical laboratory of the Kintampo Health Research Centre using standard staining and examination techniques. Parasites were counted against 200 white blood cells and slides declared negative only after 100 fields had been examined. Two microscopists independently read slides while discordant slides were read by a third microscopist. The agreement between two of the microscopists was taken as the final result. All microscopists were blinded to the results of RDTs. Due to logistic constraints, PCR analyses were limited to cases where there were conversions from RDT negative to positive during follow-up. All the RDTs used in the study were stored between 21°C and 35°C, consistent with the recommendations of the manufacturer. Blood culture was done using BACTEC blood culture system, with subculture of positive vials and gram staining for identification.

The clinical laboratory at KHRC participates in the external quality assessments schemes provided by the College of American Pathologist, UK National External Quality Assessment Scheme (UK-NEQAS) and the National Institute for Communicable Diseases in South Africa.

### Data management and analysis

All data were double-entered in a database and verified/cleaned using FoxPro version 6. Analyses were performed using STATA version 10. The results of microscopy were used as reference to assess the accuracy and reliability of RDT results. Treatment outcomes were assessed on the basis of (1) changes in RDT result (from that obtained on day 0) within the 28-day follow-up period and the correlation with clinical outcome, (2) frequency of unscheduled visits, (3) caregiver perception of clinical outcome, (4) occurrence of fever (axillary temp>37.5°C) and (5) the reduction (from baseline) in haemoglobin level. Caregiver perception of clinical outcome was assessed on the basis of the condition of the child as reported by the caregiver during follow-up visits. Caregiver perception was considered favourable when improvement in the condition of the child relative to the time of initial presentation was reported and unfavourable when the condition was reported to be “unchanged”, “worsened” or with “new illness”. The treatment outcomes were compared between the two groups. However, given that the natural history and treatment outcomes of malaria and non-malaria fevers are likely to be inherently different, the relative risks of various outcomes were not calculated.

### Ethical considerations

Written informed consent was obtained from all caregivers before enrolment. The protocol for the study was approved by the Institutional Ethics Committee of the Kintampo Health Research Centre, the Ethics Review Committees of the Ghana Health Service and the London School of Hygiene and Tropical Medicine. The study was conducted in accordance with the principles of Good Clinical Practice. The study is a component of the trial registered in the clinical trials registry under NCT00832754.

## Results

**Figure 1:**
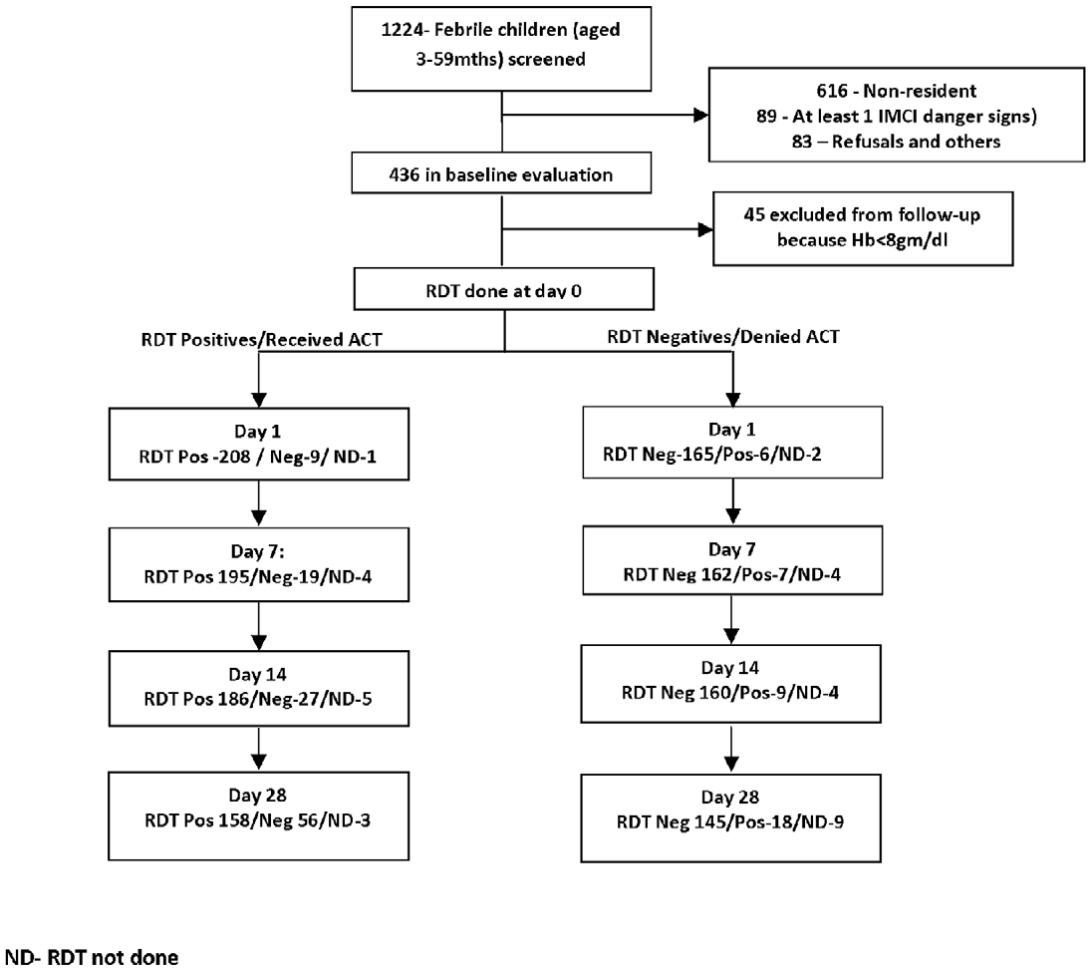
Profile of children attending and outcome of rapid test for malaria.

A total of 1224 children aged 3 months −59 months were screened and 436 children were enrolled (Figure 1) The reasons for exclusion were residence beyond 15 km from the hospital (616), presenting with at least one IMCI danger sign (89) and refusal to participate (83). All excluded children were treated using the standard treatment guidelines. Of the 436 that were enrolled, 45 had haemoglobin less than 8 gm/dL and were excluded from protocol-based clinical assessment and follow-up. They were however included in the assessment of the accuracy of RDT at day 0.

The mean age was 25.4 months (S.D. = 14.6) and only 44.3% of the children had fever (axillary Temp>37.5°C) at the time of enrolment. The children who had a RDT positive result were slightly older, more likely to have axillary temperature>37.5°C (P<0.01) and anaemic compared to children who had a RDT negative result (P<0.01). No child in the RDT negative group had malaria parasitaemia. The geometric mean parasite density was 6861/ul (range 31–1,518,575/ul). (Table 1)

Blood culture was positive for 37 (9.5%) out of 391 children, and the isolates included *Staphylococcus aureus* (24), Non-typhoid Salmonella (8), *E.coli* (1), *S. pneumonia* (1), *Acinetobacter sp* (1), *Proteus sp* (1) and *Pseudomonas sp* (1). Although a slightly higher proportion of RDT-negative children had a positive culture than RDT-positive children (11.6% versus 7.8%; p = 0.37), the difference was not statistically significant (Table 1).

**Table 1:** Demographic and baseline clinical characteristics of children.

Variable		RDT Positive	RDT Negative	P-value
Sex	Male	143 (54.4%)	93 (53.8%)	0.09
	Female	120 (45.6%)	80 (46.2%	
Age (months)	Mean (s.d.)	26.8 (14.0)	23.1 (15.1)	0.01
	≤12mths	54 (20.5%)	57 (33.0%)	
	>12mths	209 (79.5%)	116 (67.1%)	
Temperature (C)	Mean (s.d.)	37.8 (1.3)	37.0 (0.9)	<0.01
Fever at presentation	Yes	152 (57.8%)	41 (23.7%)	
Hemoglobin (gm/dl)	Mean (s.d.)	9.6 (1.9)	11.3 (1.3)	<0.01
Blood Smear	Positive	199 (75.7%)	0.0 (0.0%)	<0.01
Bacterial growth	Positive	17 (7.8%)	20 (11.6%)	0.37
Clinical diagnosis at enrolment	ARI	113 (51.8%)	135 (78.0%)	<0.01
	Diarrhoea	31 (14.2%)	35 (20.2%)	0.12
	Skin infections	27(12.4%)	28 (16.2%)	0.28
Category of drugs prescribed	ACT	217 (99.5%)	0 (0.00%)	<0.01
	Analgesic	207(95.0%)	166 (96.0%)	0.64
	Antibiotics	135 (61.9%)	162 (93.6%)	<0.01

### Diagnosis at presentation

Acute respiratory infection (ARI) (63.4%), malaria (55.5%), diarrhoea (16.9%) and skin infections (14.1%) were the leading diagnosis established among the 391 children at enrolment. About a third (28.6%) of them had both ARI and malaria. A higher proportion of RDT-negative children had ARI at enrolment than in RDT-positive children (78.0% versus 51.8%; p<0.01). There were no statistically significant differences in the proportion of children presenting with diarrhoea or skin diseases between the RDT-negative and RDT-positive children (Table 1). Unspecified acute febrile illness was the diagnosis in 8.1% of RDT-negative children.

### Antibiotic prescription

A higher proportion of RDT-negative children were given antibiotics than RDT-positive children (93.6% vs 61.9%; p<0.01). Children diagnosed with ARI (95.2% vs 42.7%: p<0.01), skin diseases (87.3% vs 74.1%; p = 0.07) or diarrhoea (80.3% vs 75.1%: p = 0.37) were more likely to be prescribed an antibiotic. However, those diagnosed with either malaria (61.8 % vs 97.3%: p<0.01), or unspecified acute febrile illnesses (57.1% vs 76.7%: p = 0.09) were less likely to be prescribed an antibiotic.

### Accuracy and reliability of CareStart

**Figure 2:**
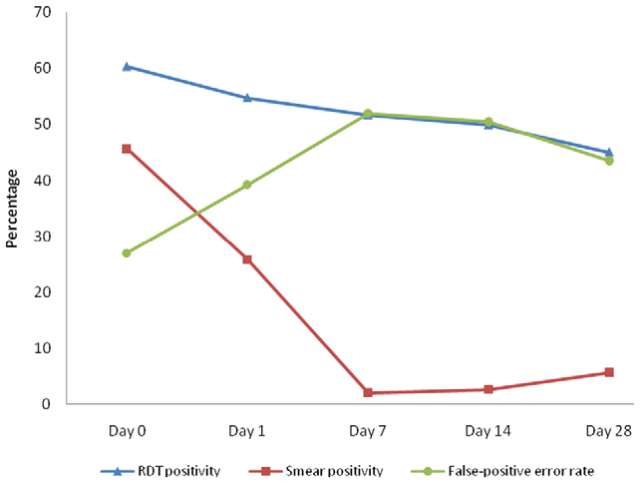
Proportion of children positive for malaria by RDT and blood smear microscopy and false positive error rates of RDT on day 0 and during scheduled follow-up days.

The sensitivity of CareSart for the diagnosis of malaria on day zero was 100.0%. Specificity was however 73.0% (95% C.I. 67%–78%) while positive and negative predictive values were 75.7% (95% C.I. 70%–81%) and 100% respectively. The false positive error rate at day zero was 27% (95% C.I. 19%–30%). Over the 28-day follow-up period, the rate of RDT conversion from positive to negative was markedly slower than blood smear parasite clearance. As a result, RDT false positive error rates were over 50% by day 7 and 14, and 43.5% on day 28 post-treatment (Figure 2). However, children presenting with fever and having a positive RDT result during the scheduled follow-up days were 16.7% on day 1, 3.5% (day 2), 5.6% (day 7), 5.2% (day 14) and 10.6% (day 28).

### Changes in RDT results and correlation with clinical outcome

Over the 28-day follow-up period, 32 (18.5%) children who tested RDT negative on day 0, converted to RDT positive. The conversions were 6 (3.5%), 7 (4.0%), 8 (5.2%) and 11 (10.4%) on days 1, 7, 14 and 28 respectively. All 32 were microscopy and PCR negative on day 0. In 7 (4.0%) of these children, conversion to RDT positive was associated with fever (axillary temperature>37.5C), giving a malaria incidence of 0.53 per person year. In 5 of them, the children were presented on unscheduled visits, while in the remaining 2, the caregivers reported “new illness” on their day 28 visit. In all 7 cases, conversion to RDT positive was confirmed by positive microscopy and they were diagnosed with uncomplicated malaria. No case of severe malaria or death was recorded.

### Unscheduled visits

Forty (10.2%) children made an unscheduled visit during the 28-day follow up period as a result of caregiver's perception of adverse outcome. A higher proportion of children with non-malaria illnesses made unscheduled visits on account of febrile illness than children diagnosed with malaria (13.3% vs 7.8%). The sensitivity, specificity and positive predictive value of RDT for the diagnosis of malaria on these unscheduled visits were 100%, 65.5% and 47.3% respectively. Diagnoses other than malaria made at these unscheduled visits included ARI (47.5%) and diarrhoea (10.0%). In 6 (15.0%) cases, both malaria and acute respiratory infections were diagnosed.

### Caregiver perception of clinical outcome

No caregiver reported a worsening of the condition of the child compared to the status at enrolment. However, the proportion of caregivers who reported the emergence of “new illness” or “unchanged” condition increased from 5.1%, (day 1) to 14.7% (day 28). On all scheduled follow-up days (except on day 14), the proportion of caregivers reporting less favourable treatment outcomes was higher in the non-malaria group compared to the malaria group (Table 2).

**Table 2:** Caregivers' perception of clinical outcome in RDT negative (non-malaria) and RDT positive (malaria) children.

Day of visit	Caregiver perception	RDT negative (non-malaria)*	RDT positive (malaria)*
**Day 1**	Unsatisfactory	10 (5.8%)	10 (4.6%)
	Satisfactory	163	208
**Day 2**	Unsatisfactory	17 (9.8%)	12 (5.5%)
	Satisfactory	156	206
**Day 3**	Unsatisfactory	17 (9.8%)	9 (4.1%)
	Satisfactory	156	209
**Day 7**	Unsatisfactory	13 (7.5%)	15 (6.9%)
	Satisfactory	160	203
**Day 14**	Unsatisfactory	16 (9.3%)	24 (11.0%)
	Satisfactory	157	194
**Day 28**	Unsatisfactory	30 (17.4%)	27 (12.4%)
	Satisfactory	142	190

* Column percentages

### Fever clearance (temperature>37.5 C)

The proportions of children having fever were 6.1%, 2.3%, 1.8%, 1.5%, 2.6% and 4.6% on days 1,2,3,7, 14 and 28 post-treatment respectively. On day 3, a higher proportion of children in the non-malaria group had fever than children in the malaria group (2.89% versus 1.82%). On all scheduled follow-up days, a higher proportion of children in the non-malaria group had temperature above values recorded on day 0 (Table 3).

**Table 3:** Change in body temperature (from day 0) in RDT negative (non-malaria) and RDT positive (malaria) children.

Day of visit	Direction of temperature change	RDT negative (non-malaria)*	RDT positive (malaria)*
**Day 1**	Increase	37 (21.6%)	19 (8.9%)
	Same or decrease	134	194
**Day 2**	Increase	32 (18.7%)	20 (9.2%)
	Same or decrease	139	197
**Day 3**	Increase	31 (17.9%)	16 (7.3%)
	Same or decrease	142	202
**Day 7**	Increase	23 (13.4%)	18 (8.3%)
	Same or decrease	149	199
**Day 14**	Increase	32 (18.8%)	15 (6.9%)
	Same or decrease	138	201
**Day 28**	Increase	28 (16.8%)	16 (7.4%)
	Same or decrease	139	200

* Column percentages

### Post-treatment change in haemoglobin

**Figure 3:**
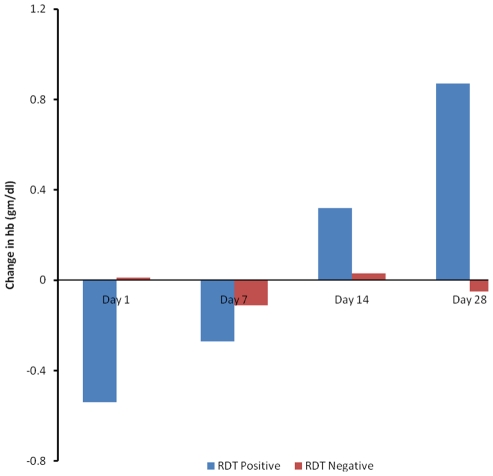
Mean change in haemoglobin (from day 0) for RDT positives (malaria) and negatives (non-malaria) children.

The proportions of children with haemoglobin lower than that recorded at enrolment were 61.1%, 52.1%, 39.9% and 32.7% on day 1, 7, 14 and 28 post-treatment respectively. The proportion of children having a reduction in Hb level on day 1 post treatment compared to day 0 was higher in the malaria group than in the non-malaria group (46.8% vs 72.4%). In contrast, the proportion of children having lower Hb levels at day 28 post-treatment compared to day 0 Hb levels was higher in the non-malaria group than the malaria group (44.8% versus 23.4%) (Table 4). On average, children who had non-malaria illnesses experienced a negative change in haemoglobin at day 28 post-treatment than children who had malaria illness (−0.05 versus +0.87) (Figure 3).

**Table 4:** Changes in haemoglobin (from day 0) in RDT negative (non-malaria) and RDT positive (malaria) on scheduled follow up days.

Day of visit	Direction of change	RDT negative (non-malaria)*	RDT positive (malaria)*
**Day 1**	Decrease	79 (46.8%)	157 (72.4%)
	Same or increase	90	60
**Day 7**	Decrease	79 (46.5%)	121 (56.5%)
	Same or increase	91	93
**Day 14**	Decrease	76 (45.2%)	76 (35.7%)
	Same or increase	92	137
**Day 28**	Decrease	73 (44.8%)	50 (23.5%)
	Same or increase	90	163

* Column percentages

## Discussion

This study was primarily powered to test the accuracy of the RDT and the treatment outcomes were secondary end-points. The comparative analyses of treatment outcomes were exploratory and should be interpreted with caution. The findings will require further investigation in appropriately-powered studies. Notwithstanding these limitations however, a pattern of comparatively unfavourable outcomes in children in the non-malaria group emerges that raises concern about their recovery when managed using current guidelines, and possibly the safety of restricting ACT to RDT positive cases in high malaria transmission setting. First, the RDT negative to positive conversion rate of 18.5% over a 28–day period appears high when compared to 0.8% found in a cohort of children (average age of 5years) in a study in a region of low malaria transmission in Uganda. [30] The malaria incidence of 0.53 per person year (extrapolated from a 28–day follow-up period) was also higher than the 0.42 and 0.45 that were observed in the Ugandan study and in another study in south-central Tanzania [31] where transmission is relatively lower than in Kintampo. Unlike in the Uganda study however, ours and the Tanzanian study did not have background cohorts (outside of the febrile children enrolled in the study) against which the general incidence of malaria over the same period could be compared. In the Uganda study, the incidence of malaria in the seven days following a negative smear in a febrile patient was significantly lower than the incidence of malaria over the entire course of the study (0.42 vs. 1.01 per person year, p = 0.0003). However since the risk of malaria is unlikely to be uniform over the period after a febrile episode, it is conceivable that a longer period of follow-up would have witnessed increased cases of malaria, particularly in the non-malaria group that was denied ACT on day 0 because their RDT results were negative. In a cohort of children in Uganda, 50% of children who had asymptomatic parasitaemia become symptomatic malaria within 30 days [32]. This has similarly been found in studies in other sub-Saharan African countries [33].

If previous IMCI guidelines (antimalarial for all fever cases) had been applied in these cohorts of children however, nearly all of them would have been given an antimalarial. For those who did not have malaria (RDT negatives), such antimalarial would have offered some prophylactic benefit that would have conferred some protection from malaria for possibly 4–12 weeks depending on the half-life of the partner antimalarial in the ACT that is given. On the other hand unnecessary ACT use would have exposed the partner drug to selective mutation of falciparum resistance strains. [34]

In the Ugandan and Tanzanian studies [30], [31] as well as our study, all the cases of malaria that occurred during the follow-up period among initially RDT-negative children were identified early and managed before the development of complications. We believe however that the assurance of safety this provides is misleading as the measures instituted in these studies to ensure follow-up of the children cannot be replicated under routine conditions. The children enrolled in the studies were selected using criteria that maximised the possibility of follow-up and close monitoring e.g. living within a defined distance of the facility. In the real-life setting, the policy of restricting ACT to RDT-positive will be applied irrespective of where in the community the child will be coming from, and some children with false negative malaria diagnosis may report late to the facility for review and could develop severe disease.

The decision to deploy test-based management of malaria therefore needs to be carefully evaluated, within the context of local levels of malaria transmission. In high-transmission settings the risk of malaria and delayed treatment could outweigh the advantages of targeted treatment. Health worker perceptions that children who are denied ACT tend to return to the clinic sooner, would undermine the confidence with which they adhere to the revised test-based treatment guidelines.

In Tanzania, a study among severely-ill children (2 months – 13 years) that used different brands of HRP-2 RDT found sensitivities levels of between 96.4% and 98.2%. [35] Critically important were the false-negative results among patients in whom levels of parasitaemia were in excess of 2000/microL, a phenomenon similarly observed in a study in Ethiopia where RDT missed three cases (out of 34) in which parasitaemia were between 40–60,000/microL. [36] In both instances, genetic heterogeneity of the HPR-2 expression was considered as a possible explanatory factor. Although the clinical outcome of patients in this category was not reported in both studies, generally the clinical consequences of such false-negative results, particularly in a population of severely-ill children could be profound. Exploring the reasons for false-negative results, in the presence of high-levels of parasitaemia should be a research priority. This includes the occurrence of the prozone effect [37], [38], [39] and or microscopically false-negative results.

The over-emphasis on malaria has been at the expense of diagnosis and effective treatment of other causes of childhood fevers in malaria high transmission settings. [26], [40] Consequently, very little is known about the definitive diagnosis and outcome of the management of non-malaria febrile illnesses. In this study, care was standardized using IMCI-based case management guidelines. However the pattern of less favourable outcomes in children with non-malaria illnesses suggests that care for these children through this strategy was probably less than optimal.

While a comparison on the basis of giving (malaria cohort) or denying ACT (non-malaria cohort) has been presented, it is important to acknowledge that the two groups of children represent different pathologies. For this reason, it has been suggested that statistical testing of observed differences between the two groups is not appropriate, and observed trends in the malaria cohort cannot be used as a comparator against which to assess trends in the non-malaria cohort. [31]. However we believe this position is arguable for the following reasons: (1) Observed differences between the two cohorts could be explained by factors other than differences in underlying pathologies alone e.g. attitude and or competence of attending health workers, appropriateness of current guidelines, availability of appropriate diagnostic tools (2) The pattern of treatment outcome in non-malaria illnesses in malaria endemic areas is less well-studied and no standards are currently available to define the state or time point of improvement (3) the pattern of treatment outcome in malaria is well-described and likely to form a part of the expectations of health workers and caregivers. It is conceivable that the pattern of less favourable outcome (in the non-malaria cohort) may have been present even in the era of presumptive malaria treatment but did not attract attention because almost all fevers were considered as malaria. As the policy of test-based malaria management continues to be rolled out, the challenge of how to appropriately manage non-malarial febrile illnesses would become important. This would include how caregivers perceive the outcome of care for children who are denied ACT on account of RDT-negative tests. Test-based management of malaria will more clearly delineate non-malarial febrile illnesses but improved guidelines and better diagnostics will be required to guarantee appropriate management of such cases. [14], [26]

The 9.5% rate of positive blood culture among out-patient children in this study is lower than rates of between 21.3% and 26% among hospitalised patients in two studies in Kumasi, Ghana. [41], [42], [43]. It is however similar to rates obtained among hospitalised children in Tanzania (9.4%) and Kenya (7.1%). [44], [45] As in the present study, non-typhoid Salmonella and *S. aureus* were the major organisms isolated as in the Kenyan and Tanzanian studies. This contrast to the findings of some other studies in the same countries where bacteraemia rates among outpatients were much lower and other organisms other than non-typhoid Salmonella and *S. aureus* dominated. [44], [45]

Although the RDT used in this study showed a perfect sensitivity of 100%, in about 1 out of 4 cases, the positive results on day 0 were false positives. Misleading interpretation of such positive RDT results have the potential to lead to the withholding of life-saving antibiotics. [46] The persistence of HRP-2 antigen in the blood leading to high false-positive results during follow-up is consistent with well-documented characteristics of HRP-2 based RDTs [24], [47], [48] 41 A false-positive error rate of over 50%, two to three weeks after an index event highlights the need for targeted training for clinicians in the correct interpretation and application of RDT results. The false positive RDT results due to the persistence of HRP-2 antigen can be overcome with PLDH based RDTs. However the co-incidence of malaria parasitaemia along with other infections is more complex to address in high transmission settings. Economic analyses that make the case that using RDT is cost-effective must take into account the quantity of ACTs that would be wasted in such situations. The deployment of RDTs in test-based malaria management should be accompanied by intensive clinical training that emphasise the message that using RDT does not preclude the need for full clinical assessment in the management of childhood febrile illnesses.

Two studies funded by the ACT Consortium (www.actconsortium.org ) that are specifically-designed to address the question of safety in restricting ACT to RDT-positive cases in under-five children in high and low transmission settings are currently underway. The findings of these studies will further clarify the issues raised in this study.

### Conclusions

The brand of RDT tested has good sensitivity and specificity. However a minority of children who will not receive ACT based on RDT result may develop clinical malaria within a short period in high transmission settings. This could undermine caregivers' and health workers' confidence in the new guidelines. Improving the quality of management of non-malarial febrile illnesses should be a priority in the era of test-based management of malaria.

## Supporting Information

Protocol S1 **Trial Protocol.**
(DOCX)
