# Supplementary material for: Accuracy of Rapid Tests for Malaria and Treatment Outcomes for Malaria and Non-Malaria Cases among Under-Five Children in Rural Ghana
Source: PLoS One. 2012 Apr 13;7(4):e34073. doi: 10.1371/journal.pone.0034073 (PMC3325982; doi:10.1371/journal.pone.0034073)
Supplement: Protocol S1 — Trial Protocol. (DOCX) [file pone.0034073.s001.docx]

Title

**Accuracy of rapid tests for malaria and treatment outcomes for malaria and non-malaria cases among under-five children in rural Ghana**

Investigators

**Frank Baiden**, Malaria Group, Kintampo Health Research Centre, Kintampo, Ghana

**Jayne Webster**, Department of Disease Control, London School of Hygiene and Tropical Medicine, London, United Kingdom

**Mathilda Tivura**, Malaria Group, Kintampo Health Research Centre, Kintampo, Ghana

**Rupert Delimini**, Malaria Group, Kintampo Health Research Centre, Kintampo, Ghana

**Yvonne Berko**, Malaria Group, Kintampo Health Research Centre, Kintampo, Ghana

**Seeba Amenga-Etego**, Computer Centre, Kintampo Health Research Centre, Kintampo, Ghana

**Akua Agyeman-Budu**, Clinical Laboratory, Kintampo Health Research Centre, Kintampo, Ghana

**Akosua B Karikari**, Microbiology Department, University of Development Studies, Tamale

**Jane Bruce**, Department of Disease Control, London School of Hygiene and Tropical Medicine, London, United Kingdom

**Seth Owusu-Agyei**, Directorate, Kintampo Health Research Centre, Kintampo, Ghana

**Daniel Chandramohan**, Department of Disease Control, London School of Hygiene and Tropical Medicine, London, United Kingdom

**SUMMARY**

**(maximum 250 words):**

Prescribing antimalarial drugs on the basis of clinical judgement results in over use of antimalarials and inappropriate treatment of febrile illness due to other cause such as pneumonia. The introduction of artesunate based combination therapy (ACT) for malaria warrants accurate diagnosis of malaria in order to maximise the benefits of ACT. The practice of clinical judgement based diagnosis of malaria is in part due to the non-availability of microscopy to confirm malaria. The availability of rapid diagnostic tests (RDT) for malaria has opened the possibility of confirming malaria even in remote health facilities. However effective use of RDT and ACT depends on the accuracy and safety of RDT based treatment practices and on factors related to the health delivery system. Furthermore there is evidence that offering intermittent treatment for malaria can reduce the incidence of malaria and anaemia in young children. Thus we propose to study the accuracy and safety of RDT based diagnosis and treatment of febrile illness, health system determinants of effective use of RDTs and the public health outcomes of RDT based ACT for malaria.

In stage I, the accuracy of RDT and the consequences of false positive and negative diagnosis with RDT will be studied in a cohort of 400 febrile <5 year old children; and the delivery system determinants of effective use of RDT will be assessed through structured observation and in-depth interviews of health care providers and users.

In stage II, a cluster randomised trial of RDT based versus clinical judgment based treatment of febrile illness on the incidence of malaria in <48 month old children will be conducted. Thirty health centres will be randomly allocated to RDT based treatment or clinical judgment based treatment arm and one hundred <2 year old children from the catchment area of each health centre will be followed for 2 years. The cost effectiveness of RDT based approach will be compare with the clinical judgement based treatment.

**^[[1]](#footnote-2)^b. Stage I - component A: Accuracy of RDT and the outcome of treatment based on RDT results**

**b.1 Research questions**

***Primary***

- 1. What is the sensitivity and specificity of Paracheck cassettes in Ghana to diagnose malaria?

***Secondary***

- 1. What is the sensitivity and specificity of paracheck cassettes to diagnose low parasite density malaria?
  2. What is the sensitivity and specificity of microscopy to diagnose malaria?
  3. What are common infectious agents in RDT+ and RDT- patients?
  4. What is the prognosis of participants who had a false +ve or –ve diagnosis of malaria?

**b.2. Study site and popualtion**

A prospective cohort study will be conducted at the Kintampo District Hospital (KDH). A cohort of suspected malaria cases of children (< 5 years of age) attending KDH during the study period.

**b.3 Inclusion criteria**

1. suspected cases of uncomplicated malaria; (2) willing to take part in the study; (3) lives within 15 km radius of KDH

**b.4. Exclusion criteria**

(1) severe malaria; (2) severe anaemia (Hb<8g/dl); (3) severe malnutrition

**b.5. Enrolment and follow up**

Children attending KDH with suspected malaria will be screened for the exclusion and inclusion criteria. An informed written consent will be obtained from caretakers of those children who fulfil the inclusion and exclusion criteria and enrolled in the study. At enrolment contact details, demographic, socio-economic and clinical data will be obtained using a structured case record form (CRF). A venous blood sample (5ml) will be collected at enrolment to test for malaria (RDT, thick and thin blood film, and filter paper samples for PCR) and for other common infections in Ghana (pneumonia, influenza, meningitis, typhoid). The RDT positive cohort of participants (RDT+ve) will be treated with AS+AQ according to the national guidelines and if there are signs suggestive of other co-morbidities they will be treated with appropriate medicines in addition to AS+AQ. The RDT negative cohort (RDT-ve) will be treated with an appropriate drug regimen according to the clinical diagnosis. Both RDT+ve and RDT-ve cohorts will be visited at homes on day 1, 2,3, 7, 14, and 28 post treatment to assess the progress of resolution of clinical symptoms and signs. A finger prick sample will be collected on day 7, 14, and 28 post treatment for RDT, blood slide and PCR from all participants. In addition if the clinical condition deteriorates at any time during the post treatment period a finger prick blood sample will be collected for blood slides, RDTs and PCR. Participants with a positive blood slide for malaria parasites at post treatment day 7, 14 or 28 will be treated with quinine. Participants developing severe disease at any time during the follow up period will be transferred to the hospital for further investigation and management, and their treatment outcomes will be followed until day 28 post treatment. Participants who do not recover by day 28 post treatment will be followed for extended period as required.

**b.6. Study procedures and follow up chart**

| **Procedures** | **D0** | **D1** | **D2** | **D3** | **D7** | **D14** | **D28** |
| --- | --- | --- | --- | --- | --- | --- | --- |
| Screening | X |  |  |  |  |  |  |
| Informed consent | X |  |  |  |  |  |  |
| Completion of enrolment form | X |  |  |  |  |  |  |
| Clinical evaluation | X | x | X | x | x | X | X |
| Blood samples for RDT, Blood Slide and PCR | X |  |  |  | x | X | X |
| Venous blood samples for culture and serology | X |  |  |  |  |  |  |
| Completion of follow up form |  | x | X | x | x | X | X |

**b.7. Laboratory procedures**

RDTs will be performed as per the instructions of the manufacturer. Blood smears will be stained with 10% Giemsa for 15 minutes and read by two independent microscopists blinded to the RDT results for speciation, and quantification. Parasite density will be estimated by counting parasites against 200 WBC. A blood smear will be considered negative if no asexual forms were seen after observing 500 WBC. Discordant results (33% difference in quantification or positive/negative results) will be read by a third microscopist; agreement between any two micoscopists and the average parasite density will be deemed to be the correct reading. Genotyping of malaria parasite using PCR will be done at KDH. Blood culture for bacteria and Widal test for typhoid will be conduced at Noguchi . The serology for influenza will be conducted in a WHO prequalified centre. Since there is no WHO accredited laboratory to test influenza serology in Ghana we will obtain Material Transfer Agreement before transferring blood samples to any laboratory outside Ghana. Since these laboratories are far away from the study sites the results of these investigations may not be available for clinical management of children.

**b.8. Endpoints/outcome variables**

The primary endpoint is the sensitivity and specificity of RDTs compared to PCR. The secondary endpoints are (1) sensitivity and specificity of RDTs for malaria parasite density 100 to 500/µl; (2) sensitivity and specificity of microscopy; (3) fever clearance time; (4) symptoms and signs clearance time (number of days required to clear the symptoms and signs that were present at enrolment)

**b.9. Sample size**

The primary end point for estimating the sample size is the sensitivity of RDTs to diagnose malaria. We assume that the RDTs should have at least 95% sensitivity to be useful in Ghana. To estimate a sensitivity of 95% with a 95% confidence limit of +/- 3%, 203 true positive cases of malaria will be needed. Since the blood samples for RDT, blood slides and PCR will be collected at enrolment there is no risk of loss to follow up and there will be more PCR positive cases than RDT positive and therefore we estimate that 200 cases of RDT positive malaria will be adequate. Similarly this sample size is adequate to determine the sensitivity of microscopy compared to PCR.

The symptoms and signs clearance time will depend on the underlying cause of illness and therefore likely to vary between RDT+ cohort (predominantly malaria) and RDT- cohort (most non-malaria fever). Since the proportion false negative and false positive cases of malaria is small the differences in the symptoms and signs clearance time between the two groups cannot be attributed to the inaccuracy of RDTs. However this parameter is useful to understand the symptoms and signs resolution pattern of RDT+ and RDT-ve cases. We assume that the proportion of participants clearing fever by day 3 post treatment will be 95% in the RDT+ cohort and 85% in the RDT-ve cohort. The loss to follow up by day 3 will be <5%. A study with 80% power to detect this difference between the two cohorts with 95% significance will need around 200 participants in each cohort.

The proportion of participants developing severe disease during the follow up period will be few and therefore the study will have very limited power to make comparison between the two cohorts.

**b.10. Data analysis**

To estimate the sensitivity and specificity of RDTs and microscopy for diagnosing malaria, the PCR results will be used as the gold standard. A comprehensive analysis plan will be developed at a later stage to address all research questions.

**b.11.Ethics**

All study procedures will be clearly explained to participants while obtaining informed consent. We will provide standard health care according to the current national treatment protocol to all study participants. In addition we will follow up the participants more closely and frequently in order to minimise and unexpected adverse outcomes. Ethical approval of the protocol will be obtained from the Institutional Review Board of Kintampo Health Research Centre, Ghana Health Service National ethics Committee and the London School of Hygiene & Tropical Medicine ethics committee.

1. This component is part of the larger project that - Effects of restricting the use of Artesunate plus amodiaquine combination therapy to malaria cases confirmed by a dipstick test: A cluster randomised control trial. **Reference ClinicalTrials.gov Identifier:: NCT00832754** [↑](#footnote-ref-2)
